# Dynamics of paediatric urogenital schistosome infection, morbidity and treatment: a longitudinal study among preschool children in Zimbabwe

**DOI:** 10.1136/bmjgh-2017-000661

**Published:** 2018-03-27

**Authors:** Derick Nii Mensah Osakunor, Takafira Mduluza, Nicholas Midzi, Margo Chase-Topping, Masceline Jenipher Mutsaka-Makuvaza, Theresa Chimponda, Enwono Eyoh, Tariro Mduluza, Lorraine Tsitsi Pfavayi, Welcome Mkululi Wami, Seth Appiah Amanfo, Janice Murray, Clement Tshuma, Mark Edward John Woolhouse, Francisca Mutapi

**Affiliations:** 1 Centre for Infection, Immunity and Evolution, Institute of Immunology and Infection Research, University of Edinburgh, Edinburgh, UK; 2 Department of Biochemistry, College of Health Sciences, University of Zimbabwe, Harare, Zimbabwe; 3 School of Laboratory Medicine and Medical Sciences, University of KwaZulu Natal, Durban, South Africa; 4 Department of Medical Microbiology, College of Health Sciences, University of Zimbabwe, Harare, Zimbabwe; 5 Centre for Immunity, Infection and Evolution, Usher Institute of Population Health Sciences and Informatics, University of Edinburgh, Edinburgh, UK; 6 National Institute of Health Research, Ministry of Health and Child Care, Harare, Zimbabwe; 7 Epidemiology and Disease Control, Ministry of Health and Child Care, Harare, Zimbabwe; 8 NIHR Global Health Research Unit Tackling Infections to Benefit Africa (TIBA), University of Edinburgh, Edinburgh, UK

**Keywords:** dynamics, incidence, morbidity, paediatric, praziquantel efficacy, prevalence, preschool, schistosomiasis, Zimbabwe

## Abstract

**Background:**

Recent research has shown that in schistosome-endemic areas preschool-aged children (PSAC), that is, ≤5 years, are at risk of infection. However, there exists a knowledge gap on the dynamics of infection and morbidity in this age group. In this study, we determined the incidence and dynamics of the first urogenital schistosome infections, morbidity and treatment in PSAC.

**Methods:**

Children (6 months to 5 years) were recruited and followed up for 12 months. Baseline demographics, anthropometric and parasitology data were collected from 1502 children. Urinary morbidity was assessed by haematuria and growth-related morbidity was assessed using standard WHO anthropometric indices. Children negative for *Schistosoma haematobium* infection were followed up quarterly to determine infection and morbidity incidence.

**Results:**

At baseline, the prevalence of *S haematobium* infection and microhaematuria was 8.5% and 8.6%, respectively. Based on different anthropometric indices, 2.2%–8.2% of children were malnourished, 10.1% underweight and 18.0% stunted. The fraction of morbidity attributable to schistosome infection was 92% for microhaematuria, 38% for stunting and malnutrition at 9%–34%, depending on indices used. *S haematobium*-positive children were at greater odds of presenting with microhaematuria (adjusted OR (AOR)=25.6; 95% CI 14.5 to 45.1) and stunting (AOR=1.7; 95% CI 1.1 to 2.7). Annual incidence of *S haematobium* infection and microhaematuria was 17.4% and 20.4%, respectively. Microhaematuria occurred within 3 months of first infection and resolved in a significant number of children, 12 weeks post-praziquantel treatment, from 42.3% to 10.3%; P<0.001.

**Conclusion:**

We demonstrated for the first time the incidence of schistosome infection in PSAC, along with microhaematuria, which appears within 3 months of first infection and resolves after praziquantel treatment. A proportion of stunting and malnutrition is attributable to *S haematobium* infection. The study adds scientific evidence to the calls for inclusion of PSAC in schistosome control programmes.

**Key questions**

**What is already known about this topic?**

- Epidemiological studies indicate that preschool-aged children (PSAC) aged ≤5 years are exposed to schistosome infection, the consequences of which manifest later in life.
- Unlike school-aged children, there are no longitudinal studies tracking the dynamics of new infections, the development of morbidity and implications on current and future health in this age group.

**What are the new findings?**

- We determined for the first time levels of schistosome morbidity in PSAC attributable to *Schistosoma haematobium* infection, that is, 92% of microhaematuria, 38% of stunting and depending on what index is used, 9%–34% of malnutrition. We recorded significant annual incidence of new schistosome infection (17.7%) and urinary morbidity (microhaematuria; 20.4%) with significant quarterly incidences.
- We showed that microhaematuria occurred within 3 months of first infection and resolved after praziquantel (PZQ) treatment.
- We indicated that a significant amount of morbidity, as measured by microhaematuria, resolved within 3 months of effective treatment with PZQ (significant reduction from 42.3% vs to 10.3% (P<0.0001)).

**Recommendations for policy**

- The findings indicate that schistosome morbidity in PSAC can be reversed by PZQ treatment.
- The findings contribute to the scientific evidence base for prioritising schistosome treatment in PSAC, to reduce infection and morbidity and promote child health and development.

## Introduction

Of the 123 million children worldwide affected with schistosomiasis, about 50 million are preschool-aged children (PSAC), that is, ≤5 years.^1^ Nonetheless, schistosome infection and morbidity dynamics in this age group are less characterised compared with school-aged children (SAC), that is, ≥6 years. For example, there are several studies describing and quantifying schistosome-related morbidity including haematuria, nutritional deficiencies and delayed growth and cognition in SAC^2 3^ but no comparable comprehensive studies in PSAC.

Epidemiological studies in PSAC clearly indicate that infection occurs in early childhood,^4–7^ and if untreated, the infection can lead to health consequences later in life.^8^ Despite this importance of childhood infections, there is a paucity of longitudinal studies tracking the dynamics of new first infections, the development of morbidity and implications on current and future health in this age group. The definition of schistosome pathology and morbidity continues to be refined in attempts to better characterise clinical manifestations, for example, as with female genital schistosomiasis,^9^ and to identify applicable morbidity markers of disease, for example, urine albumin–creatinine ratio.^10^ Growth and nutrition-related morbidities associated with schistosomiasis have also only recently become more widely recognised.^11^ There is therefore a need to collate all of this new knowledge to better define schistosomiasis in PSAC, where manifestations of disease are poorly described.^6^

In this study, we aimed to describe the baseline dynamics of schistosome infection and morbidity in Zimbabwean PSAC exposed to *Schistosoma haematobium*. A cohort of schistosome-negative children was followed for a year to document infection and morbidity incidence, as well as the effects of treatment on infection and morbidity. This study investigates the ability of existing diagnostic and morbidity tools to quantify and monitor early infection and morbidity. It also contributes to disease burden estimates and the dynamics of infection and morbidity in PSAC. This knowledge will inform the design and implementation of interventions targeted at this age group.

## Methods

### Consent

Permission to conduct the study in the province was obtained from the Provincial Medical Director. Prior to enrolment, the study aims and procedures were explained to all participants and their parents/guardians in English or in the local language, Shona. Written informed consent was obtained from the participants’ parents/guardians as appropriate. Recruitment into the study was voluntary and parents/guardians were free to withdraw the participants at any time with no further obligation.

### Study site and period

The study was conducted in 13 villages in the Shamva district, northeast of Zimbabwe (17°10′0″S 31°40′0″E) from February 2016 through to February 2017. This is one of seven districts in the Mashonaland Central province of Zimbabwe, whose people are primarily subsistence farmers. There is a cold dry (April–July), hot dry (August–October) and rainy season (November–March).^12 13^ The area was selected for this study on urogenital schistosomiasis because the prevalence of *S haematobium* is high (>50%), while the prevalence of *Schistosoma mansoni* and soil-transmitted helminths is low (<15%).^14^

### Study design

This study was part of a larger longitudinal parasitological and immunological project, following the treatment-reinfection study design widely used in human helminth field studies. There was a cross-sectional study at baseline, followed by a 1-year longitudinal study. Recruited children were screened at baseline for schistosome infection and morbidity to describe the epidemiology of infection and morbidity in this population. The larger study is comparing the impact of regular quarterly screening and treatment (group 1) and biennial screening and treatment (group 2). This study reports on the findings in the children screened quarterly (group 1). Following the baseline recruitment, age and sex-matched *S haematobium*-negative children who fulfilled the inclusion criteria were randomly allocated into groups 1 and 2. A total of 1783 children were invited to participate, of which 1502 provided samples for parasitological diagnosis at baseline. After allocation to the two groups of the study, 525 children who were schistosome-negative by egg count, provided a blood sample for serological assays and consented to participate in the longitudinal follow-up, formed the group 1 cohort, which was followed up every 3 months to detect new schistosome infections by egg count, and morbidity by microhaematuria.

Children were recruited from crèches, early child development centres, and preschools. Parents/guardians of children not attending any of the educational programmes (eg, children <3 years) were invited through the community nurse and village health workers to report to the sampling centre; that is, the centre used by the community for the Expanded Program for Immunisation (eg, school or primary health centre) for enrolment into the project. A questionnaire designed in English and translated into the local dialect (Shona) was administered to gather demographic data and establish exposure behaviour.

### Study inclusion criteria

At baseline, the study enrolled children aged 6 months to 5 years who met the following inclusion criteria. Participants had to (i) be lifelong residents of the study area, (ii) have no previous antihelminthic treatment, (iii) be negative for *S mansoni* and (iv) consent to participate. To be included in the longitudinal cohort, children who had fulfilled the inclusion criteria described above had to meet an additional criterion of being diagnosed negative for *S haematobium* by egg count at baseline.

### Anthropometry

Weight (nearest 0.1 kg) and height (nearest 0.1 cm) without shoes and in light clothing was measured using an electronic scale and a stadiometer, respectively. For very young babies, height was measured with an infantometer baby board, and weight measured with a baby scale. The mid-upper arm circumference (MUAC) was measured (nearest 1 mm) using a child MUAC tape; on the left arm, midpoint between the shoulder and the tip of the elbow, with the arm relaxed and hanging down the body. Growth and nutritional status was assessed using the WHO Anthro software V.3.0.1 (http://www.who.int/childgrowth/en/).^15^ This generated Z-scores for specific measures of nutrition and growth, that is, stunting by height-for-age (HAZ), underweight by weight-for-age (WAZ) and body mass index-for-age (BAZ) and malnutrition by MUAC (MUAC and MUACZ) and weight-for-height (WHZ). Measures were considered abnormal when Z scores were <–2.^16^

### Parasitological diagnosis

About 50 mL of urine sample was collected from each participant on three successive days and a stool specimen was collected on a single day from each participant. Samples were collected between 10:00 hours and 14:00 hours, and processed within 2 hours of collection. For very young children, urine bags (Hollister 7511 U-Bag Urine Specimen Collector, Hollister, Chicago, Illinois, USA) and disposable diapers were used to collect urine and stool samples respectively. Urine samples were examined microscopically for *S haematobium* infection following the standard urine filtration method^17^ and the number of eggs was reported per 10 mL of urine. Stool samples collected were processed using the Kato–Katz method^18^ and parasite eggs enumerated under a light microscope for *S mansoni* (in duplicates) per gramme of stool.

Children were diagnosed positive for helminth infection if at least one parasite egg was detected in their urine or stool samples. All children who were positive for *S haematobium* infection were treated with a single dose of praziquantel (PZQ) at the standard 40 mg/kg bodyweight at each visit. Tablets were crushed and administered with squash and sliced bread^19^ by local nurses. A post-treatment efficacy check (egg count) was carried out for all such participants at each subsequent follow-up (12 weeks post treatment).

### Detection of urinary morbidity

Urine samples collected were examined for visible haematuria (macrohaematuria). Microhaematuria was determined by dipping the reagent end of Uristix reagent strips (Uripath, Plasmatec, UK) into fresh, well-mixed urine for 40 s and the test area compared with a standard colour chart as per manufacturer’s instructions. The strength of the colour change indicates varying concentrations of blood present in the sample, that is, negative, trace, positive (+), positive (++), positive (+++) and positive (++++). For analysis purposes, microhaematuria was classified as either negative or positive.

### Sample size calculations

The sample size used for this study was based on the larger, longer-lasting study comparing reinfection rates following different treatment regimens, of which this is a subset. Calculations were based on previous studies showing that PZQ treatment reduces reinfection rates by at least 50%.^5 14^ Statistical power analysis was performed using Gpower V.3.1.5.^20^ Based on an expected prevalence of 6.7%, as derived from our previous study in children aged 1–5 years,^21^ a sample size of 214 in each group would provide α=0.05 and power=0.80. Allowing for a 20% dropout rate, the sample size required for each group during follow-up was 256. The sample size for the larger reinfection study, that is, 525 in a group, was therefore sufficient for this aspect of the study.

### Statistical methods

Data analyses were performed using SPSS V.22 (IBM) and GraphPad Prism V.7.02 (GraphPad Software). The χ^2^ (or Fisher’s exact test for small sample sizes) and the Mann-Whitney was used to test for differences in categorical and numerical variables, respectively. Infection intensity for *S haematobium* was defined as the arithmetic mean egg count/10 mL of at least two urine samples collected on three consecutive days. The egg count data was further log-transformed (log_10_ [egg count+1]) to meet the normality assumption of parametric statistics. At baseline, infection intensity (log-transformed) and its relationship as a function of age was determined using a linear regression model. To determine the infection prevalence based on a binary response as a function of age, logistic regression modelling was used as previously described.^22^ The effect of different factors on the prevalence of schistosome infection and morbidity was determined using logistic regression and the results reported as adjusted ORs (AORs) and 95% CI, along with the test for significance.

For a given morbidity marker, the attributable fraction (AF) in the exposed population (*S haematobium-*positive) and in the total population was calculated using Miettinen’s formulae^23^:

$$\text{AF in the exposed population}\left(AFe\right)=\frac{\left(RR-1\right)}{RR}$$

$$\text{AF in the total population}\left(AFp\right)=Pe\times AFe=Pe\times \frac{\left(RR-1\right)}{RR}$$

In the formulae, RR is the risk ratio of morbidity associated with exposure, Pe is the prevalence of morbidity among the exposed (*S haematobium-*positive). Because the AFs in this case are from a helminth study and estimated at the cross-sectional level, we substituted the RR with prevalence ratios (PR) as recommended in helminth epidemiology.^24^ The PR was estimated as a ratio of the proportion of infected individuals with morbidity to the proportion of uninfected individuals with morbidity. AFs were calculated on morbidity markers with PR>1, suggesting an increased risk of morbidity from schistosome infection.^24^ Treatment efficacy was assessed by means of egg reduction rates (ERR) and cure rates (CR) as described previously.^21^

Approximate CIs were calculated using the modified Wald method^25^ and P<0.05 was considered significant.

## Results

### Demographics

Of the 1502 recruited into the study, 794 (52.9%) were male. Age range was between 0.5 and 5 years (median=3.5 years; IQR 2.5–4.3). The youngest participant in whom *S haematobium* infection was detected was a year old. Maximum loss to follow-up was recorded at first follow-up in May 2016 (64 participants; 12.2%). Overall follow-up rates in the longitudinal cohort, including participants for post-treatment efficacy check, were 87.8% in May 2016, 93.7% in August 2016, 95.1% in November 2016 and 88.8% in February 2017.

### *S haematobium* epidemiology at baseline

**Figure 1:**
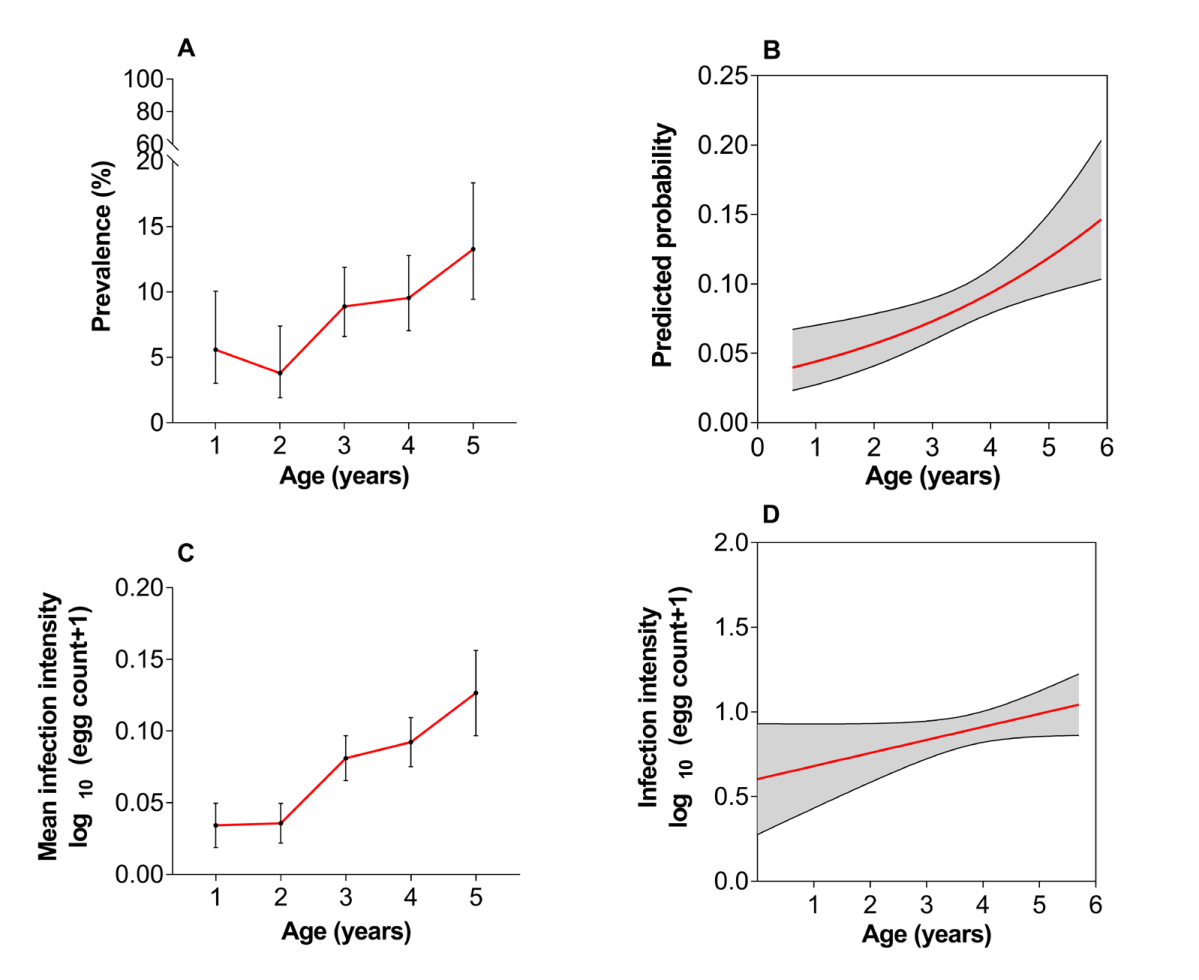
(A) *Schistosoma haematobium* infection prevalence with age; prevalence varied with age (P<0.001) and (B) age-predicted probability of infection; prevalence increased as children grew older (P=0.002). (C) *S haematobium* infection intensity with age; intensity varied with age (P<0.001) and (D) age-predicted intensity of infection; infection intensity increased as children grew older. Error bars indicate 95% CI (A) or SEM (C), and shaded areas indicate 95% CI; (B, D).

*S haematobium* infection prevalence at baseline in the 1502 participants was 8.5% (95% CI 7.2 to 10.0). The median age of children positive for infection was significantly higher compared with those negative for infection (4.0 vs 3.5 years; P=0.001). Infection prevalence increased with age as shown in figure 1A,B. The overall mean infection intensity was 7.9 eggs/10 mL urine (95% CI 6.4 to 9.7). The majority of children, 93.7% (95% CI 87.9 to 97.0) presented with light infections (<50 eggs/10 mL of urine) based on the WHO classification.^19^ Infection intensity increased with age as shown in figure 1C,D. There was no significant difference in infection prevalence between males and females; 8.9% (95% CI 7.1 to 11.1) and 8.1% (95% CI 6.3 to 10.4; P=0.067), respectively.

### Morbidity at baseline

Prevalence of urinary morbidity was 0.7% (95% CI 0.3 to 1.5) for macrohaematuria and 8.6% (95% CI 6.9 to 10.6) for microhaematuria. Malnutrition measured by different indices were as follows: MUAC, 2.2% (95% CI 1.4 to 3.2), MUACZ, 7.4% (95% CI 6.0 to 9.1) and WHZ, 8.2% (95% CI 6.8 to 9.9). Prevalence of underweight measured by WAZ was 10.1% (95% CI 8.5 to 11.9), and stunting by HAZ was 18.0% (95% CI 16.0 to 20.3). Comparing infected versus uninfected children, prevalence of microhaematuria (43.5%; 95% CI 34.8 to 52.6 vs 3.4%; 95% CI 2.4 to 5.0; P<0.001) and stunting (27.0%; 95% CI 19.9 to 35.6 vs 17.0%; 95% CI 14.9 to 19.4; P=0.009) was significantly higher among children with *S haematobium* infection.

### Morbidity attributable to *S haematobium* infection

**Figure 2:**
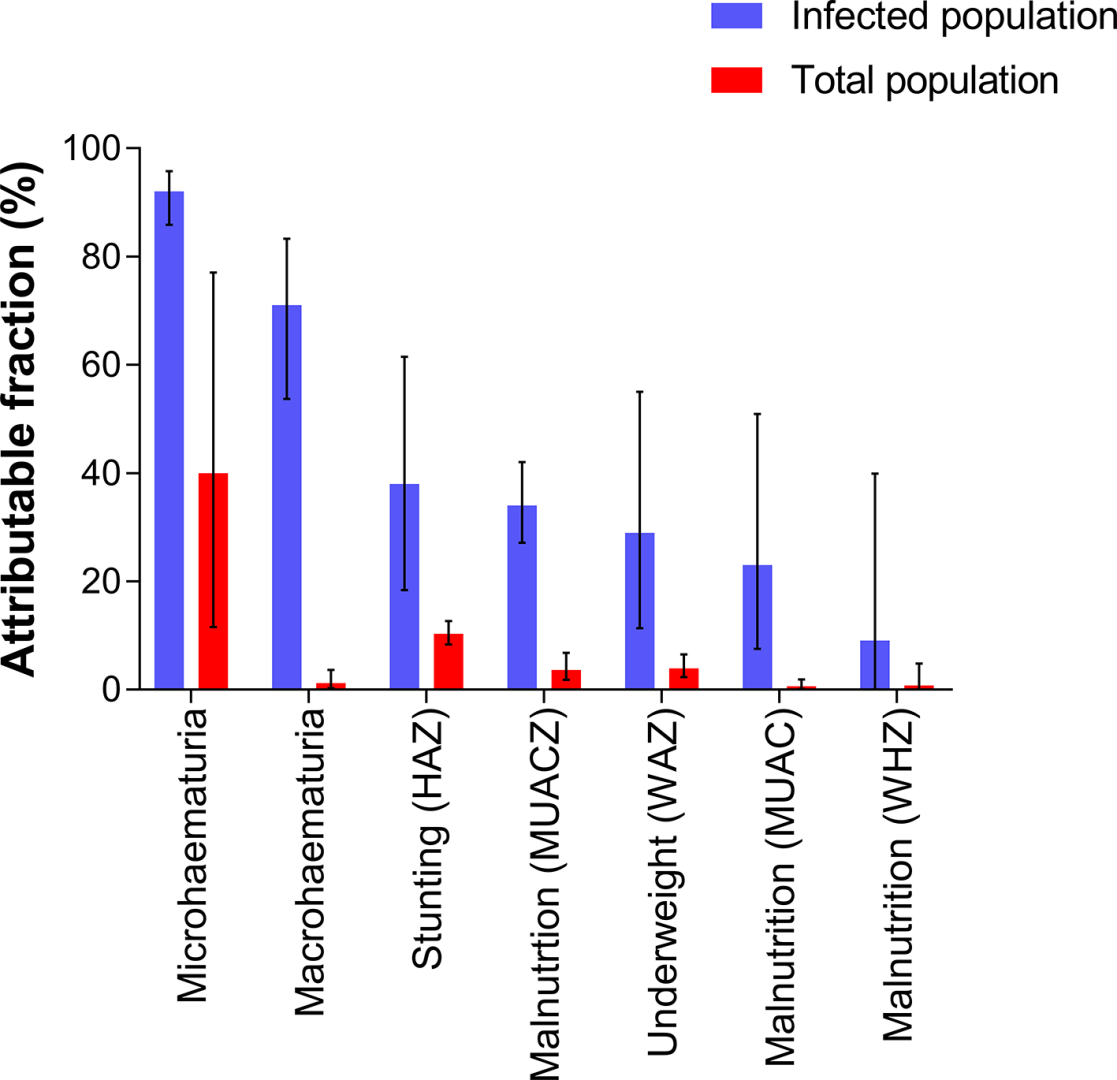
Estimated proportion of morbidity attributable to *Schistosoma haematobium* infection in the infected population (blue; AFe) and in the total population (red; AFp). Error bars indicate 95% CIs. BAZ, body mass index-for-age Z scores; HAZ, height-for-age Z scores; MUAC, mid-upper arm circumference Z scores; WAZ, weight-for-age Z scores; WHZ, weight-for-height Z scores .

Morbidities from schistosome infection are not specific and may relate to different physiological, biochemical and immunological processes. We determined how much of the detected morbidity was attributable to schistosome infection by first determining PRs. All the morbidity markers considered had PR >1 (significant association with schistosome infection) except underweight by BAZ (table 1). Based on AFs, microhaematuria was the most dominant morbidity marker attributed to schistosome infection both in infected children and at the population level. Macrohaematuria, on the other hand, was highly attributable to schistosome infection in the infected population but this was not the case in the total population. Of the anthropometric markers, stunting was the most dominant marker attributed to schistosome infection both at the population level and among the infected children (figure 2).

**Table 1:** Prevalence ratios (PRs) for detected schistosome-related morbidity

Morbidity	Diagnostic tool	PR (95% CI)
Microhaematuria	Urine dipsticks	12.6 (11.6 to 14.1)
Macrohaematuria	Visual inspection (colorimetry)	3.4 (1.9 to 5.4)
Stunting	HAZ	1.6 (1.05 to 2.31)
Malnutrition	WHZ	1.1 (0.9 to 1.4)
	MUACZ	1.5 (1.3 to 1.9)
	MUAC	1.3 (0.8 to 1.9)
Underweight	WAZ	1.4 (1.2 to 1.6)
	BAZ	1.0 (0.8 to 1.3)

BAZ, body mass index for age Z scores; HAZ, height-for-age Z scores, MUAC, mid-upper arm circumference Z scores; WHZ, weight-for-height Z scores; WAZ, weight-for-age Z scores.

### Likelihood of schistosome infection and morbidity

**Figure 3:**
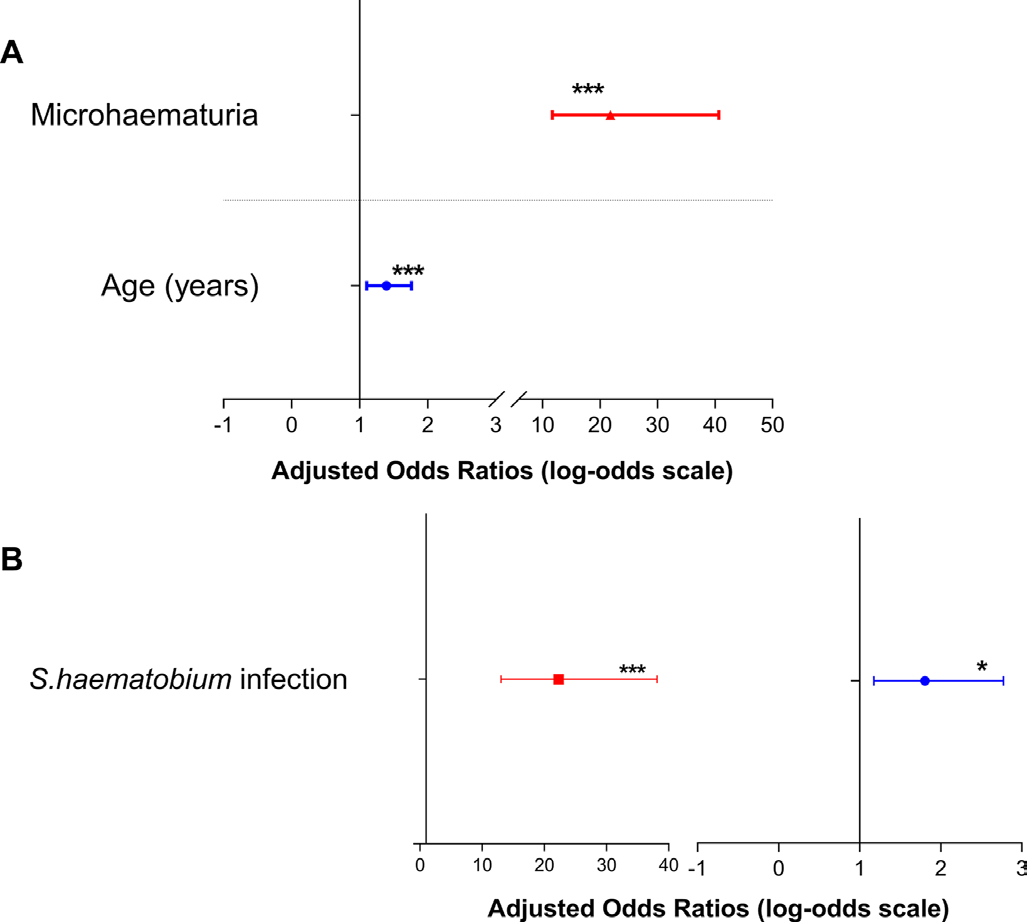
Forest plot showing (A) the odds of presenting with *Schistosoma haematobium* infection and (B) odds of presenting with microhaematuria (left) and stunting (right). Error bars indicate the 95% CIs. *P<0.05, ***P<0.001. Non-significant variables were excluded from the final logistic regression model.

Multiple logistic regression analysis showed that with every unit increase in age, children were more likely to acquire *S haematobium* infection (AOR=1.4; 95% CI 1.1 to 1.8; P=0.005). Children who presented with microhaematuria were more likely to be positive for *S haematobium* infection (AOR=21.8; 95% CI 11.7 to 40.7; P<0.001) as shown in figure 3A. Similarly, children presenting with *S haematobium infection* were more likely to present with microhaematuria (AOR=25.6; 95% CI 14.5 to 45.1; P<0.001) and stunting (AOR=1.7; 95% CI 1.1 to 2.7; P=0.014) as shown in figure 3B.

### Incidence of infection and morbidity

To determine infection and morbidity incidence, 525 schistosome-negative children were followed quarterly for 12 months to determine schistosome infection and morbidity acquired in the previous three months. Based on the longitudinal data, annual incidence of *S haematobium* infection was 17.4% (95% CI 13.7 to 21.8) and that of microhaematuria was 20.4% (95% CI 15.8 to 26.0). *S haematobium* incidence rates in the dry season was 4.9% in May (95% CI 3.1 to 7.8) and 6.5% in August (95% CI 4.1 to 9.9) while that in the rainy season was 3.8% in November (95% CI 2.1 to 6.6) and 3.7% in February (95% CI 2.1 to 6.5). Difference in overall incidence rates however was not significant between the dry (10.4%; 95% CI 7.6 to 14.1) and rainy seasons (7.4% total; 95% CI 5.0 to 10.9; P=0.175). The quarterly incidence of microhaematuria recorded was 2.0% in May (95% CI 0.4 to 5.9), 2.8% in August (95% CI 1.0 to 6.6), 13.3% in November (95% CI 9.6 to 18.3) and 4.3% in February (95% CI 2.2 to 8.1).

### Treatment efficacy and effects on morbidity

The treatment efficacy was calculated from children positive for infection at baseline and those that became infected throughout the year. Thus, a total of 187 children were treated for infection (127 at baseline and 60 from the longitudinal cohort), of which post-treatment data were available for 156 (follow-up rate: 83.4%). PZQ was efficacious in reducing *S haematobium* infection, as indicated by the high CR (96.2%; 95% CI 91.7 to 98.4) and ERR (99.8%; 95% CI 99.2 to 100). In addition, the mean infection intensity pretreatment (7.1 eggs/10 mL urine; 95% CI 5.9 to 8.6) was significantly reduced at post-treatment follow-up (1.1 eggs/10 mL urine; 95% CI 1.0 to 1.2; P<0.001).

To determine the effects of treatment on morbidity identified at baseline, data for microhaematuria were available for 78 of the 127 S *haematobium*-positive cases identified. Within this cohort, 42.3% (95% CI 32.0 to 53.4) were positive for microhaematuria and this declined significantly post-treatment (10.3%; 95% CI 5.1 to 19.2; P<0.001).

**Figure 4:**
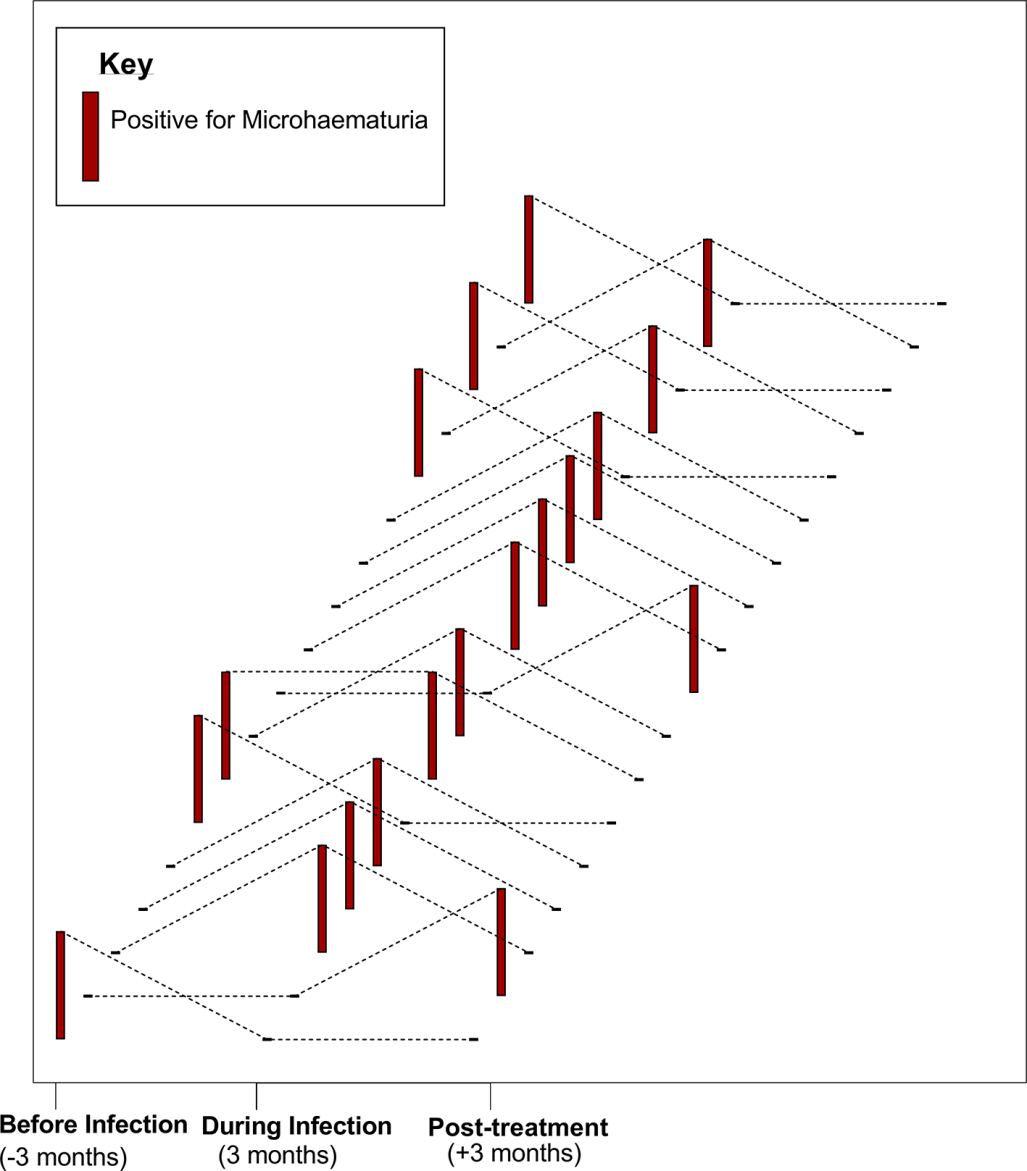
Impact of *Schistosoma haematobium* infection and praziquantel (PZQ) treatment on morbidity (microhaematuria). Microhaematuria status for 18 individual participants is shown at three time points: before, during and post infection (post treatment). Each data set (dotted line) represents one individual. Tall, red bars indicate positive microhaematuria, and a black dash indicates negative microhaematuria at specific time points.

A pooled analysis of participants in whom new infections were detected throughout the follow-up period (group 1) was done to determine the dynamics of microhaematuria with infection, before, during and post infection. A total of 60 new infections were detected throughout the follow-up period. Of this, microhaematuria was detected among 18 individuals; 6 (33.3%) preinfection, 11 (61.1%) during infection and 2 (11.1%) post treatment. In 61.1% of these individuals, microhaematuria coincided with the detection of *S haematobium* infection (within 3 months) and had resolved by the next survey at 3 months post treatment of infection (figure 4).

## Discussion

Contrary to the previously held assumption of low risk to schistosome infection in PSAC,^26^ the frequency of schistosome infections among infants and young children is being increasingly recognised.^27^ We conducted a longitudinal study in a cohort of Zimbabwean PSAC to determine the prevalence, dynamics and incidence of first urogenital schistosome infection and morbidity and its associated risks and health impacts. We showed that PSAC present with schistosome infection (estimated by egg count) and associated morbidity (determined by microhaematuria, growth and nutritional markers) from an early age. We also found that schistosome infection and morbidity can be detected early in PSAC using parasitology and microhaematuria within 3 months of infection and is resolved after treatment.

The observed baseline prevalence of *S haematobium* infection in PSAC (8.5%) is comparable to levels recorded in PSAC in Ghana (11.2%),^4^ Malawi 10.7%^28^ and in our recent studies in Zimbabwe, that is, 13.5%^10^ and 6.7%.^21^ PSAC however present with light infections^22 27 29^ and parasitological egg counts underestimate the prevalence of schistosome infection.^4 22^ We anticipate this prevalence to increase if the more sensitive serological diagnostic tools are used.^22^ In agreement with previous findings,^7 22 30–32^ infection prevalence and intensity increased as children grew older.

The majority of morbidity biomarkers associated with schistosomiasis are non-specific and relate to various physiological, biochemical and immunological processes.^33^ We determined the prevalence of morbidity and how much of this was attributable to *S haematobium* infection. Microhaematuria was the most dominant marker for schistosome-related morbidity, and children with *S haematobium* infection were more likely to present with microhaematuria and vice versa. This agrees with our previous findings in Zimbabwe^10^ and that by researchers in Nigeria^34^ on the significance of microhaematuria as a point of care field marker of morbidity in PSAC.

In addition to biomarkers, we also investigated the prevalence of stunting and malnutrition in the children. To the best of our knowledge, this is the first study to show the relationship between *S haematobium* infection and chronic growth failure (Stunting by HAZ) in PSAC, although studies on polyparasitism^35 36^ and few schistosome-specific studies^37 38^ have documented this effect in older children. In accordance with our findings, stunting as detected in older children and adolescents is believed to be the result of chronic antiparasite inflammation which persists during childhood.^39^ Causality is difficult to establish in this case due to the lag time between the initial infection and the time at which we measured growth failure, and the impact of confounding factors including diet and coinfections. However, there is the need for longer-term studies investigating the impact of treatment on growth and development measures in PSAC. Statistical modelling suggests that with early, repetitive treatment of infection before 6 years of age ‘catch-up growth’ can be effectively facilitated.^40^

While baseline prevalence and intensity of schistosome infection have been described in PSAC from several African countries, there has not been an incidence study published to date. Here, we document the incidence of urogenital schistosome infection and morbidity in PSAC. Our quarterly incidence is an indication of new schistosome infections in PSAC in endemic areas and the applicability of current tools (urine filtration and urine dipstick) to screen for early infection and morbidity. The incidence of microhaematuria and the AF analyses suggest that even in the very first episodes of infection PSAC suffer morbidity, reflected as microhaematuria; an indication of active bladder and ureteral lesions^41 42^ and blood loss even in mild schistosome infection.^43^

PZQ, the antihelminthic of choice for treatment of schistosomiasis, is safe and efficacious in PSAC.^44^ Our results 12 weeks post treatment showed that a single standard dose of PZQ was effective against *S haematobium* infection. This is consistent with reports on the efficacy of PZQ treatment for schistosomiasis in PSAC.^5^ Microhaematuria correlates with *S haematobium* infection,^42 45^ and treatment with PZQ reduces morbidity (microhaematuria) as used in large-scale chemotherapy for SAC.^33 46^ We observed that microhaematuria occurred rapidly within 3 months of exposure to infection and resolved within 3 months after treatment with PZQ. This is an important indicator for non-delayed schistosome screening and treatment in PSAC, to avert cumulative morbidity which can affect overall health.^1^ Observation from our field studies prove that suggestions to empower health workers to screen for infection, and making PZQ available in health centres for treatment on detection will be an important control strategy in this age group.^47^

The seasonal pattern of infection incidence detected is in agreement with the fact that during the dry seasons snail vectors and larval schistosomes become concentrated at permanent and slow-moving water sources, increasing the risk of infection.^48^ Our observation from fieldwork also indicates that during the rainy seasons, households are less reliant on water sources for chores and children are less likely to visit water bodies for recreational purposes.

The impact of schistosome infection on the health of children is likely to be greater than those explored here, for example, its impact on neurocognitive development. Mechanistic and epidemiological studies separating the effects of schistosome infections from other confounders would be informative in identifying and portioning causation in AFs. The present study did not measure the impact of existing feeding, nutrition habits and socioeconomic status on stunting and its relationship to schistosome infections. A limitation of the parasitological detection of infection is that some light infections may have been missed, resulting in underestimation of the infection rates observed. Nonetheless, the study allows comparison with other studies while parasitological methods remain the predominant schistosome diagnostic in PSAC.

## Conclusions

We demonstrated for the first time the incidence of schistosome infection and morbidity in PSAC. We have also shown that a significant proportion of stunting and malnutrition is attributable to *S haematobium* infection. Morbidity assessed by microhaematuria occurs rapidly within 3 months of first infection and resolves post treatment. More importantly for childhood health and development, schistosome treatment leads to a significant decline in microhaematuria and this resolution occurs within 3 months of PZQ treatment. The study adds scientific evidence to the calls for inclusion of PSAC in schistosome control programmes. Non-delayed schistosome screening and treatment in PSAC is essential to avert accumulative morbidity which can affect overall health.
